# Alcohol reduces aversion to ambiguity

**DOI:** 10.3389/fpsyg.2014.01578

**Published:** 2015-01-15

**Authors:** Tadeusz Tyszka, Anna Macko, Maciej Stańczak

**Affiliations:** Department of Economic Psychology, Kozminski UniversityWarsaw, Poland

**Keywords:** alcohol, risk aversion, ambiguity aversion, gender differences

## Abstract

Several years ago, Cohen et al. (1958) demonstrated that under the influence of alcohol drivers became more risk prone, although their risk perception remained unchanged. Research shows that ambiguity aversion is to some extent positively correlated with risk aversion, though not very highly (Camerer and Weber, 1992). The question addressed by the present research is whether alcohol reduces ambiguity aversion. Our research was conducted in a natural setting (a restaurant bar), where customers with differing levels of alcohol intoxication were offered a choice between a risky and an ambiguous lottery. We found that alcohol reduced ambiguity aversion and that the effect occurred in men but not women. We interpret these findings in terms of the risk-as-value hypothesis, according to which, people in Western culture tend to value risk, and suggest that alcohol consumption triggers adherence to socially and culturally valued patterns of conduct different for men and women.

## INTRODUCTION

Several years ago, Cohen et al. (1958) demonstrated that drivers became more risk prone under the influence of alcohol. Surprisingly, however, the drivers’ risk perception remained unchanged. This pattern suggests that the increase of risk acceptance when under the influence of alcohol is not an effect of changes in perceptions of outcomes’ probabilities, but rather is caused by a change in the evaluation of outcomes’ attractiveness. We might, then, ask how an increase in the attractiveness of an outcome would occur.

Actually, it is suggested by several studies that it is an increase in outcome desirability rather than an increase in perceived feasibility of the outcome that is responsible for heightened propensity to take risk under the influence of alcohol. For example, Sevincer and Oettingen (2014) showed that alcohol intoxication resulted in an increase of participants’ desirability (incentive value), but not feasibility of important goals. Similarly, Lane et al. (2006) found that alcohol increased individuals’ sensitivity to consequences (gains and losses), but not expectancy updating rate. Steele and Josephs (1990) explain changes in behavior under the influence of alcohol by referring to cognitive processing impairment. They claim that alcohol leads to “myopia” i.e., narrowing of the attention and focusing on the most salient features of the situation. According to the authors in real life risky situations (sexual behavior, dangerous driving, etc.) salient cues concern gains, while the likelihood of losses is less silent. However, our explanation of these results as well as that by Cohen et al. (1958) is different, motivational rather than cognitive.

One significant reason for a change may be associated with the fact that in many social contexts risk itself is considered to be of value. Indeed, in most cultures courage is considered a virtue. In line with this argument, Brown (1965) formed the hypothesis that moderate risk is valued in Western culture and that people shift toward risky decisions to gain approval from other members of their group. According to this hypothesis, people would also tend to perceive themselves to be more risk seeking than their peers. Following this hypothesis, Levinger and Schneider (1969) found that college students considered higher levels of risk to be more admirable than those levels that they had accepted in their own previous decisions. Thus, the findings of Cohen et al. (1958) could be interpreted as indicating that alcohol consumption triggers adherence to socially and culturally valued patterns of conduct, and leads to a real-life increase in willingness to take risks.

Ellsberg (1961) described a phenomenon known as ambiguity aversion. Ambiguity aversion differs from risk aversion. Risk aversion refers to the preference of having a less than expected value of a lottery for sure than the lottery itself. Ambiguity aversion refers to the preference for situations containing precisely defined probabilities of possible states of nature over situations involving undefined probabilities. Research shows that ambiguity aversion is to some extent positively correlated with risk aversion, though not very highly (Einhorn and Hogarth, 1986; Camerer and Weber, 1992).

The main question addressed in the present research is whether alcohol reduces not only risk aversion but also ambiguity aversion. Based upon the previous finding that increased risk acceptance under the influence of alcohol is not an effect of a change in perceptions of probability of outcomes, but rather an effect of a change in evaluations of attractiveness of outcomes, we hypothesized that *under the influence of alcohol people will not only be less risk averse, but will also be less ambiguity averse*.

It should be noted that “risk as value theory,” which the current hypothesis is based on, uses a concept of risky behavior in the colloquial sense referring to courage in decision making under uncertainty. Contrary to its name, it does not refer specifically to the situation of risk (understood as a combination of probabilities and outcomes), but rather broadly to choices under conditions of uncertainty, ambiguity or risk. Thus, consequently, our hypothesis says that under the influence of alcohol people will choose the option less certain but more attractive when it comes to potential outcomes.

Another question addressed in the present research concerns a possible gender difference in alcohol’s influence on ambiguity aversion. There is substantial evidence that women and men differ in risk taking (Byrnes et al., 1999; Cross et al., 2013). Moreover, stereotypically, similarly, to competitiveness and dominance, risk taking is considered to be a masculine trait (as measured by Bem, 1974). For example, Wilson and Daly (1985) concluded from their literature review that risk taking is a central characteristic of the psychology of men. Thus, assuming that the increase of risk acceptance under the influence of alcohol results from risk being valued in Western culture, we formed the hypothesis that *alcohol decreases ambiguity aversion more in men than in women*.

## MATERIALS AND METHODS

### PARTICIPANTS

One hundred participants, 46 women and 54 men, took part in the study. Their ages ranged from 18 to 43, with mean age *M* = 26.3 years, SD = 5.35 years. Most participants (*n* = 66) were educated to university degree level, 33 declared a high school education, and 2 declared lower than a high school education.

### TASK AND PROCEDURE

The study was conducted individually in a restaurant which was a part of a large leisure center^1,2^ . It was carried out in the evenings between 9 pm and 12 pm. To obtain reliable measures of people’s blood alcohol levels, the time elapsed since having the last drink or smoking a cigarette had to be at least 20 mins. A precision Breathalyzer Alkohit X100 was used to measure blood alcohol levels. One of the experimenters approached a restaurant visitor and told them that he and the other experimenter represented a Research Centre and that they were conducting a study examining how accurately people estimate their own blood alcohol level. Then, the participant was told that as compensation for participation in the study they would be offered the possibility of winning free drinks. If a person expressed willingness to participate, they were invited to a separate room where the experiment was carried out. In the experimental room, the second experimenter executed the following procedure:

(1) Participants provided demographic information concerning gender, age and education. Then they estimated their blood alcohol level, choosing one of six intervals: 0–0.2‰, 0.2–0.5‰, 0.5–1.00‰, 1.00–1.50‰, 1.50–2.00‰, and above 2.00‰.
(2) Then the experimenter gave participants a cup of water and asked them carefully to rinse their mouth (to remove any residual alcohol).
(3) Next, participants blew into the alcoholmeter until it produced a sound signaling completion of blood alcohol measurement.
(4) Finally, participants completed a task where they could win free drinks. They saw two urns. Both had labels. On one of them the label informed them that there were 30 coupons inside, of which 15 were vouchers for one free drink to use in the bar and the other 15 were empty cards (the customer did not win anything). On the second, the label informed participants that the urn contained 30 coupons, of which some were vouchers for two free drinks to use in the bar and some were empty cards (the customer did not win anything); however, the numbers of the two types of coupons were unknown to participants. The former urn was thus an unambiguous urn, offering a 50/50 chance of winning a free drink, and the latter was an ambiguous urn, offering a chance of winning a higher prize – two drinks, but with an unknown probability of success.

Thus, we measured: subjectively estimated blood alcohol level, real (objectively measured) blood alcohol level, and the choice between risky vs. ambiguous options.

## RESULTS

Real blood alcohol level and subjectively estimated blood alcohol levels were significantly positively correlated – Spearman’s rho = 0.48, *p* < 0.001, *n* = 100. Thus, participants were moderately good at estimating their real blood alcohol levels. Choices between risky vs. ambiguous options did not differ across subjectively estimated blood alcohol levels.

**FIGURE 1:**
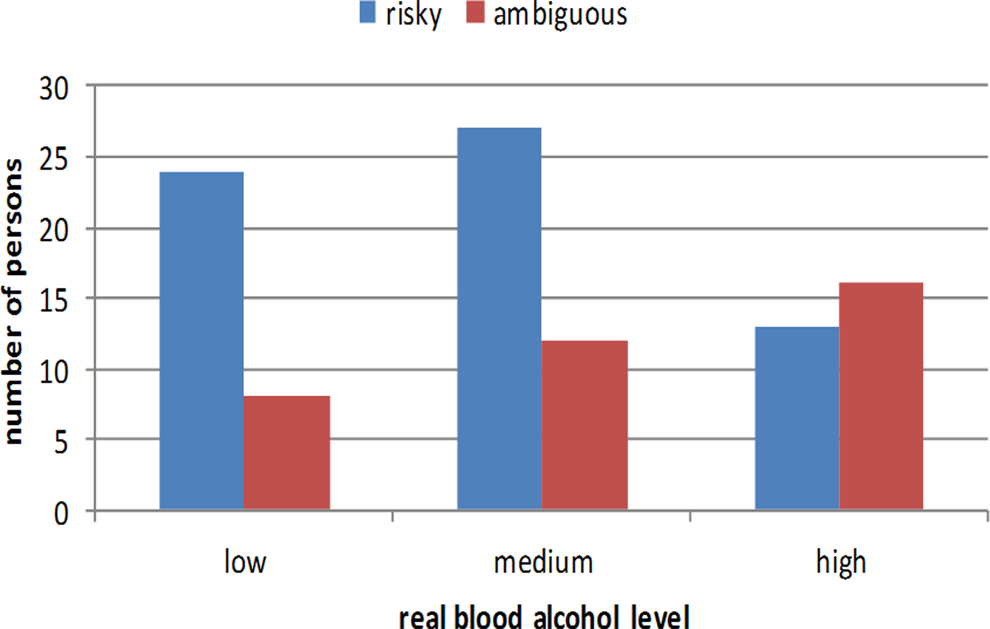
**Frequencies of choosing risky and ambiguous urns among participants with different real blood alcohol levels**.

Participants were divided into three groups depending on their real blood alcohol level: low – up to 0.5% (*n* = 32), medium – 0.51 to 1.00% (*n* = 39), and high – above 1.00% (*n* = 29). As **Figure 1** shows, there was a relationship between blood alcohol level and preferences for the risky vs. ambiguous options. Those with higher blood alcohol levels choose the ambiguous option more often than those with low alcohol levels, χ^2^ (2, *n* = 100) = 6.77, *p* = 0.03.

**FIGURE 2:**
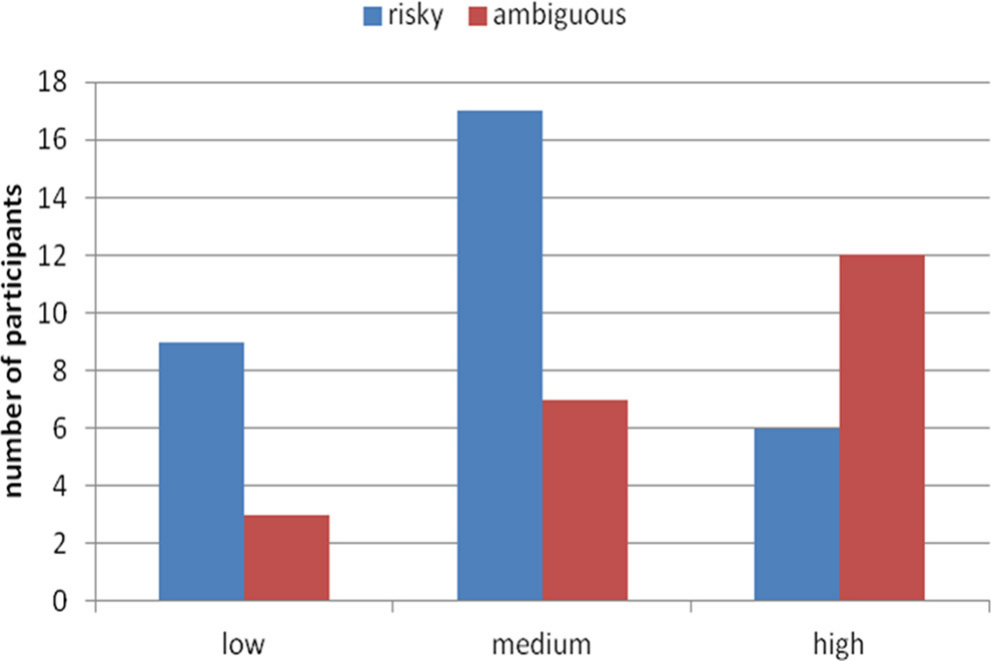
**Number of male participants with low, medium, and high real blood alcohol levels choosing the risky and ambiguous urns**.

**FIGURE 3:**
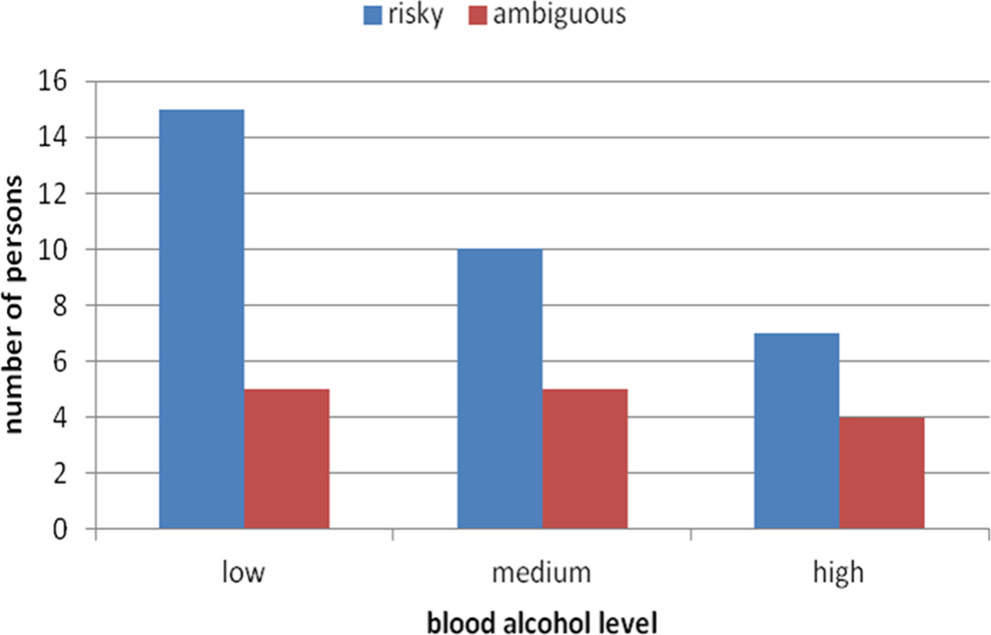
**Number of female participants with low, medium, and high real blood alcohol levels choosing the risky and ambiguous urns**.

We compared preferences for risky vs. ambiguous options as a function of level of blood alcohol separately for women and men. As **Figure 2** shows, men who had higher levels of blood alcohol chose ambiguous option more often than those with lower alcohol levels, χ^2^ (2, *n* = 54) = 7.57, *p* = 0.02. On the other hand, as **Figure 3** shows, more women with higher levels of blood alcohol than with lower levels chose risky option, yet independent of the blood alcohol level similar number of women decided for ambiguous option. Thus, blood alcohol level did not change women’s attitude toward ambiguity [χ^2^ (2, *n* = 46) = 0.52, *p* = 0.77]. This difference cannot be ascribed to the level of blood alcohol in men and women. The average level of blood alcohol was indeed slightly higher in the men than in the women sample (*M* = 0.89, SD = 0.50 vs. *M* = 0.73, SD = 0.43), but the difference was not statistically significant (*U* Mann–Whitney test, *U* = 993, *Z* = -1.92, *p* = 0.9). The difference in the level of blood alcohol was even slighter in the group of participants with the highest blood alcohol level – *M* = 1.35, SD = 0.31 for women and *M* = 1.48, SD = 0.37 for men (*U* Mann–Whitney test, *U* = 77, *Z* = 0.97, *p* = 0.33).

## DISCUSSION

The present study yielded two findings. First, it showed that in addition to the known tendency of people to become more risk prone when they consume alcohol, alcohol also reduces ambiguity aversion. Second, we found that the reduction of ambiguity aversion under conditions of alcohol consumption is more prominent in men than in women. We interpret these findings in terms of two presumptions. First, that alcohol consumption triggers adherence to socially and culturally valued patterns of conduct. Second, that people in Western culture tend to value risk (as suggested by the risk-as-value hypothesis). In line with this, we confirmed the hypothesis that alcohol consumption leads to more positive valuation of risk and courage, and, in effect, to more risky choices.

Surprisingly, we observed somewhat analogous results concerning willingness to engage in risky behavior in a study based on terror management theory. In a nutshell, according to terror management theory, people’s fear of death can be regulated through the maintenance of self-esteem. This in turn can be achieved by satisfying the norms of one’s culture (Pyszczynski et al., 1997). In line with this idea, Hirschberger et al. (2002) showed that mortality salience induction led men, but not women, to reveal high willingness to engage in risky behaviors. This finding seems parallel to ours: both consumption of alcohol and mortality salience induction reduce risk aversion in men but not in women. Both of these findings seem to be in line with the premise that in Western culture men are socialized to be more risk-oriented than women.

Furthermore, one could ask how alcohol consumption would influence the willingness of people to engage in other behaviors related to social values. For example, there is evidence, that women are socialized to be more caring (Gilligan, 1982). One can speculate, then, that alcohol consumption would result in the increase of nurturing behavior in women but not in men. Of course, this possibility needs separate examination.

On the other hand, it is likely that alcohol consumption has no influence on behaviors that are unrelated to social or cultural norms. In particular, alcohol should not influence attitude toward ambiguity that is unrelated to uncertainty of outcome occurrence. For example, Weber and Tan (2012) showed that ambiguity aversion occurs not only in the context of risk, but also in intertemporal choices (delivery of a package either in an exact time or within a range of dates). Since, to our knowledge, there is no social norm concerning the value of time inaccuracy, alcohol consumption should not reduce ambiguity aversion in intertemporal choices.

## Conflict of Interest Statement

The authors declare that the research was conducted in the absence of any commercial or financial relationships that could be construed as a potential conflict of interest.

## Footnotes

1 We were looking for a naturalistic setting for our experiment. Drinking bar where individuals decide themselves to consume alcohol seemed to be a convenient setting. Moreover, by conducting the study in a drinking bar we were able to avoid the so called “demand characteristics,” i.e., participants’ interpretations of the experiment’s purpose and changing their behavior to fit that interpretation. We believe that the cover story of our procedure provided participants with a convincing justification, and thus they expressed their true preferences.

2 Research was approved by the Commission of Ethics in Research at the Kozminski University.
